# Clinical covariates that improve surgical risk prediction and guide targeted prehabilitation: an exploratory, retrospective cohort study of major colorectal cancer surgery patients evaluated with preoperative cardiopulmonary exercise testing

**DOI:** 10.1186/s13741-022-00246-3

**Published:** 2022-05-26

**Authors:** Vladimir Bolshinsky, Hilmy Ismail, Michael Li, Jarrod Basto, Robert Schier, Anna Hagemeier, Kwok-Ming Ho, Alexander Heriot, Bernhard Riedel

**Affiliations:** 1 General Surgery, Peninsula Health, Frankston, VIC Australia; 2 Surgical Health Specialists, Frankston, VIC Australia; 3 Peter MacCallum Cancer Centre, Melbourne, VIC Australia; 4 Universität zu Köln Medizinische Fakultät, Koln, Nordrhein-Westfalen Germany; 5 Royal Perth Hospital, Perth, Australia

**Keywords:** Prehabilitation, CPET, Cardiopulmonary exercise testing, Functional capacity, Colorectal cancer surgery

## Abstract

**Background:**

Preoperative risk stratification is used to derive an optimal treatment plan for patients requiring cancer surgery. Patients with reversible risk factors are candidates for prehabilitation programmes. This pilot study explores the impact of preoperative covariates of comorbid disease (Charlson Co-morbidity Index), preoperative serum biomarkers, and traditional cardiopulmonary exercise testing (CPET)-derived parameters of functional capacity on postoperative outcomes after major colorectal cancer surgery.

**Methods:**

Consecutive patients who underwent CPET prior to colorectal cancer surgery over a 2-year period were identified and a minimum of 2-year postoperative follow-up was performed. Postoperative assessment included: Clavien-Dindo complication score, Comprehensive Complication Index, Days at Home within 90 days (DAH-90) after surgery, and overall survival.

**Results:**

The Charlson Co-morbidity Index did not discriminate postoperative complications, or overall survival. In contrast, low preoperative haemoglobin, low albumin, or high neutrophil count were associated with postoperative complications and reduced overall survival. CPET-derived parameters predictive of postoperative complications, DAH-90, and reduced overall survival included measures of VCO_2_ kinetics at anaerobic threshold (AT), peakVO_2_ (corrected to body surface area), and VO_2_ kinetics during the post-exercise recovery phase. Inflammatory parameters and CO_2_ kinetics added significant predictive value to peakVO_2_ within bi-variable models for postoperative complications and overall survival (*P* < 0.0001).

**Conclusion:**

Consideration of modifiable ‘triple low’ preoperative risk (anaemia, malnutrition, deconditioning) factors and inflammation will improve surgical risk prediction and guide prehabilitation. Gas exchange parameters that focus on VCO_2_ kinetics at AT and correcting peakVO_2_ to body surface area (rather than absolute weight) may improve CPET-derived preoperative risk assessment.

## Introduction

Technical advances in surgery and anaesthesia have led to colorectal cancer surgery being performed on increasingly older patients, often with extensive comorbid disease (Findlay et al., 2011). For the growing numbers of high-risk patients for whom surgery still forms the cornerstone of cancer treatment appropriate preoperative risk stratification is essential to enable clinicians and patients to formulate a shared treatment plan.

Decreased functional capacity (caused by factors such as lifestyle choice, ageing, cancer biology, and neoadjuvant chemo-radiotherapy) increases postoperative morbidity and mortality (Silver & Baima, 2013; West et al., 2014). Functional capacity is a modifiable risk factor: a Delphi survey of anaesthetists and consumers identified preoperative fitness training as a top-ten research priority in anaesthesia (Bainbridge et al., 2012; Boney et al., 2015). Most promising is that a number of recent studies report a substantial reduction in postoperative complications following targeted prehabilitation in patients with modifiable risk, including deconditioning (poor fitness levels), anaemia, and malnutrition (Bolshinsky et al., 2018). Even in elderly patients, published data demonstrated that in the limited window of optimization prior to major gastrointestinal cancer surgery, prehabilitation is feasible and that this translates into measurable benefits in pre and post-operative functional capacity and reduction in postoperative complications (Tsimopoulou et al., 2015; Jack et al., 2011; Barberan-Garcia et al., 2018; Howard et al., 2019).

Importantly, improved surgical risk prediction for postoperative morbidity and mortality will help identify patients that would benefit most from prehabilitation, but also guide informed decision-making in those patients at highest risk. Risk prediction can be aided by a thorough understanding of patients’ burden of comorbid disease (e.g. Charlson Co-morbidity Index or the American College of Surgeons National Surgical Quality Improvement Program [NSQIP] Surgical Risk Calculator (Bilimoria et al., 2013; Chang et al., 2016), preoperative blood tests (to identify modifiable risk factors such as anaemia, malnutrition, and inflammation) (Vardy et al., 2018; Chen et al., 2017; Edwards et al., 2011), and through assessment of functional capacity.

Functional capacity can be quantified using a static survey, e.g. Duke Activity Status Index (DASI), dynamic field walk tests, or the gold standard of cardiopulmonary exercise testing (CPET) (Moonesinghe et al., 2013). CPET is attractive in that in addition to objective assessment of preoperative functional capacity, it also provides diagnostic insight into the underlying cause of exercise limitation, and assists with exercise prescription.

Over the past two decades, CPET-derived gas exchange parameters have been increasingly studied for preoperative risk stratification, albeit in isolation and often with limited consideration of other preoperative parameters (Hightower et al., 2010). These perioperative CPET studies have generally focused on the kinetics of oxygen consumption (VO_2_) during the active cycling phase of CPET—namely VO_2_ at anaerobic threshold (AT), VO_2_ at peak exercise (pVO_2_), and typically dichotomised VO_2_ (ml/kg/min) at AT and at peak exercise to predict postoperative morbidity and mortality (Wijeysundera et al., 2018). Few studies have explored the kinetics of carbon dioxide elimination (VCO_2_) during the active cycling phase (CO_2_ elimination relative to minute ventilation [Ve/VCO_2_] at AT or end-tidal CO_2_ [P_ET_CO_2_] at AT) (Moran et al., 2016; Levett & Grocott, 2015), the kinetics of heart rate related to the exercise (Hightower et al., 2010) and gas exchange parameters during the recovery phases of CPET (VO_2_R, VCO_2_R) in relation to postoperative outcomes (Ackland et al., 2019).

This retrospective cohort study set out to further explore the predictive value of the following: (1) preoperative covariates of patient burden of comorbid disease (Charlson Co-morbidity Index) and/or preoperative serum biomarkers, and CPET-derived parameters of functional capacity; and (2) exploratory parameters of the recovery phase (after peak exercise) CPET-derived parameters on postoperative outcomes after major colorectal cancer surgery.

## Materials and methods

### Patients

This retrospective cohort study was approved by the institutional review board at the Peter MacCallum Cancer Centre (#16/19R). Consecutive patients who had undergone CPET prior to colorectal cancer surgery over a 2-year period (September 2013–August 2015) were identified within a prospectively maintained hospital database. Surgery type included segmental colorectal resection, proctocolectomy, abdominoperineal resection, pelvic exenteration, and cytoreduction surgery with hyperthermic intraperitoneal chemotherapy (HIPEC). Patients received standard perioperative care that included enhanced recovery after surgery (ERAS) pathways. The postoperative destination (intensive care, high dependency unit, or regular nursing floor) was determined by the clinical team at the conclusion of surgery for each case.

### Risk stratification

Preoperative risk assessment was estimated by routine clinical examination in the pre-anaesthesia clinic, with routine preoperative blood tests performed (full blood evaluation, urea, creatinine, and electrolytes) as clinically indicated, the Charlson Co-morbidity Index, and CPET derived heart rate and gas-exchange variables. A modified Glasgow Prognostic Score was created by substituting neutrophil count for C-reactive protein (which was not routinely measured), and 1 point was assigned for each neutrophil count > 7.5 × 10^9^ cells/l or albumin levels < 35 g/l. Zero points were assigned if both parameters were normal and 2 points assigned if both parameters were abnormal. The neutrophil-lymphocyte ratio (NLR) and platelet-lymphocyte ratio (PLR) were calculated as a marker of inflammation using the results obtained from the full blood evaluation panel (R Z, 2021)**.**

CPET was performed as per the Perioperative Exercise Testing and Training Society (POETTS) practice guidelines (Levett et al., 2018) within four weeks of patients’ scheduled elective surgery. Patients participating in the study were instructed not to eat or drink within 2 h of their scheduled CPET. The exercise test was conducted in five phases. Phase 1: Pulmonary function testing (sitting) to measure static lung function, including forced expiratory volume in 1 second and forced vital capacity. Phase 2: Resting phase—after applying the electrodes for a 12-lead ECG, arterial pressure cuff, pulse oximeter, and gas exchange collection mouth piece patients sat quietly for 3 min on the cycle ergometer for the collection of resting gas exchange derived data (CardiO2/CP System, Medical Graphics Corporation, USA). Phase 3: Unloaded cycling at 60–70 revolutions per minute (RPM) with no resistance for 3 min. Phase 4: Ramp protocol during which patients continue to cycle at 60–70 RPM with progressively increasing pedal resistance at a predetermined work rate (10–20 W/min) individualised to each patient’s physical strength. The test was stopped either when the patient fatigued or at the investigator’s discretion on the basis of signs or symptoms of cardiopulmonary distress. Phase 5: Recovery phase during which patients continue to pedal at 60–70 RPM with minimal resistance (20 W) for 5 min after peak exercise.

After CPET was completed, the test was analysed by anaesthesiologists accredited in CPET assessment (HI, BR), with independent crosschecking of the CPET data to ensure accuracy. Traditional perioperative CPET-derived parameters that were analysed included VO_2_ at anaerobic threshold (AT; ml/kg/min) and pVO_2_ corrected to patient body weight (ml/kg/min). Peak VO_2_ data were also corrected to patient body surface area (ml/min/m^2^). VO_2_ at AT was determined according to the POETTS guidelines, using the three-point estimate of modified V-slope, ventilatory equivalents, and increasing end-tidal partial pressure of oxygen (P_ET_O_2_) (Levett et al., 2018). Exploratory parameters that were analysed included CO_2_ exchange parameters (P_ET_CO_2_, Ve**/**VCO_2_—i.e. minute ventilation to CO_2_ production ratio, also known as ventilatory equivalents) at AT, heart (exercise and recovery phase) kinetics, and VO_2_ recovery in the first 5 min after achieving peak exercise.

### Patient characteristics and postoperative outcomes

Data was extracted from the medical records by three investigators (ML, JB, and VB) and cross-referenced. Extracted data included patient demographic, anthropometric factors, and postoperative complications. Postoperative complications were graded according to the Clavien-Dindo scoring system (Dindo et al., 2004). Using the established Clavien-Dindo scores, the sum of all postoperative complications was calculated using the Comprehensive Complication Index (CCI) to derive a score out of 100 (Slankamenac et al., 2013). A patient-centric measure, Days at Home within 90 days after surgery (DAH-90), was used to account for complications, mortality, and re-admission rates (Myles et al., 2016). This metric has been shown to be very sensitive to quality improvement initiatives (Wijeysundera et al., 2018). Patients were followed up for a minimum of two years after their surgical procedure for overall survival analysis.

### Statistical analysis

Statistical methods consisted of standard reporting of descriptive baseline statistics and standard statistical regression methods using the base package of the R language for statistical computing (version 3.6.0) and add-on packages (survminer, ggplot2, gdata, graphics, grDevices, Hmisc, R2wd, rJava, utils, and xlsx). Some parameters were scaled as indicated in order to report their hazard ratios and the associated 95% confidence interval (95% CI) on a meaningful scale and were considered both in raw form and dichotomised at critical values where it was considered clinically meaningful to do so. Univariable Cox proportional hazards regression was performed for survival analysis, with survival curves compared by Log-Rank test and shown alongside number at risk (*P* < 0.05 considered significant).

To explore improved risk prediction using multivariable models, bi-variable models were considered within this data set due to the limited number of patients and the frequency of missing data. Due to the likelihood of high co-linearity within the various CPET-derived variables and inflammatory biomarkers, only models combining one CPET variable with one inflammatory marker were considered together. The best CPET factor (by univariable *P*-value) was combined with the single most predictive inflammatory marker, being the one which adds the most predictive value to the best CPET factor (again using *P*-value as the metric). This was undertaken for each of CCI, DAH-90, and overall survival as the independent variable. Due to the exploratory nature of this study adjustment for multiplicity was not used to correct for the large number of tests performed.

For the exploratory analysis of the recovery phase parameters, serial changes in HR (HRR, heart rate recovery), VO_2_, and CO_2_ exchange parameters (P_ET_CO_2_, Ve/VCO_2_) from the time point of peak exercise through to the 5-min recovery period were analysed by repeated measures ANOVA (to avoid multiple statistical comparisons). In the univariate analyses, cases with missing data required for the analyses concerned were excluded and in the repeated measures of 2-way ANOVA and comparisons of slopes, all cases were included and only the missing data points were not considered in the overall analysis. Area-under-the-receiver-operating-characteristic (AUROC) curve was used to assess the ability of the rate (or slope) of recovery of each CPET parameter to discriminate between patients with and without mortality.

## Results

The study cohort was representative of a complex colorectal surgical practice at a quaternary cancer centre. Eighty-four consecutive patients that underwent CPET prior to major colorectal cancer surgery during a 2-year period (August 2013 and September 2015) were identified and a minimum of 2-year postoperative follow-up for overall survival was performed in this cohort. Two patients required emergency surgical intervention prior to elective surgery at an external institution and consequently the number of patients available for overall survival analysis was 82 patients, with a greater male representation (55%). Baseline patient characteristics, surgical procedures, and postoperative complications are detailed in Table 1.

**Table 1:** Baseline demographic factors, surgical procedures, and postoperative outcomes

Variable	Count (%)/value
**Preoperative variables**	
• Age (years)	62 [56–70]
• Gender (M/F)	46 (55%)/38 (45%)
• Smoker (yes/no)	11 (13%)/73 (87%)
• Charlson Co-morbidity Index	6 [3–6]
• Evidence of metastatic disease at time of surgery	32 (39%)
**Surgical procedures**	
• Rectal cancer resection	42 (51%)
• Cytoreduction and HIPEC	23 (28%)
• Abdominal multi-visceral resection	34 (41%)
• Total pelvic exenteration	14 (17%)
**Intraoperative outcomes**	
Intraoperative blood transfusion	17 (21%)
**Postoperative outcomes**	
• Re-look operative intervention during index admission	5 (6%)
• Postoperative complications (by highest Clavien-Dindo grading)	
0	25 (34%)
I–II	25 (34%)
III–IV	21 (29%)
V	2 (3%)
• Composite Complication Index	24.2 [16.1–39.7]
• DAH-90	75 [58–79]

Continous not normally distributed variables are presented as median with interquartiles range (IQR), categorical variable are presented as absolute and relative frequencies *n* (%)

*HIPEC*, hyperthermic intraperitoneal chemotherapy; *IQR*, interquartile range; *DAH*-90, Days at home within 90 days of surgery

### Complexity of surgical disease

This cohort of patients had a high burden of comorbid disease (Charlson Co-morbidity Index; median [IQR] = 6 [3–6]). Preoperative metastatic disease was documented in 39% of patients undergoing surgical intervention. More than half of the patients underwent intervention for rectal cancer, with an aggressive surgical approach. Multi-visceral resection was required in 41% of patients. One-third of the study cohort underwent cytoreduction and HIPEC. Intraoperative blood transfusion was required in 21% of patients and 5 patients required a re-look laparotomy. Two-thirds of patients suffered postoperative complications (CCI; median [IQR] = 24.2 [16.1–39.7]), and median DAH-90 was 75 (IQR, 56–79) days.

### Postoperative complications

Univariable linear regression assessed the association between Charlson Co-morbidity Index, traditional preoperative laboratory values, CPET-derived variables, and postoperative complications (assessed by the CCI; Table 2).

**Table 2:** Postoperative complications. Association between the Comprehensive Composite Index (CCI) and continuous or dichotomised preoperative variables, including the Charlson Co-morbidity score, laboratory biomarkers, and CPET-derived variables. Analysis was done using linear regression analysis

Preoperative parameters	Dichotomised reference level	***N***	beta	95%CI	***P***-value
**A. Univariable analysis**					
**Charlson Co-morbidity Index**					
***i. Continuous***		79	− 0.29	− 2.24, 1.67	0.780
***ii. Dichotomised***	< 5	79	− 1.02	− 9.67, 7.62	0.820
**Laboratory parameters**					
***i. Continuous***					
• Haemoglobin (g/l)		58	− 0.31	− 0.59, − 0.03	0.031
• White cell count (× 10^9^/l)		58	2.46	0.52, 4.40	0.013
• Neutrophils (× 10^9^/l)		58	3.17	0.87, 5.47	0.007
• Albumin (g/l)		34	− 1.29	− 2.18, − 0.41	0.004
• Modified (neutrophil-albumin) Glasgow Prognostic Score		34	12.00	4.32, 19.70	0.002
***ii. Dichotomised***	***Risk cut-point***				
• Haemoglobin (g/l)	< 110	58	− 9.39	− 21.40, 2.67	0.130
• White Cell Count (× 10^9^/l)	> 11	58	23.50	3.63, 43.30	0.020
• Neutrophils (× 10^9^/l)	> 7.5	58	26.10	8.66, 43.60	0.003
• Platelets (× 10^9^/l)	> 350	58	6.74	− 8.42, 21.90	0.380
• Albumin (g/l)	< 35	34	− 12.2	− 23.0, − 1.46	0.026
***iii. Ratios***					
• Neutrophil-lymphocyte ratio (NLR)	> 4	58	2.91	− 0.80, 13.80	0.600
• Platelet-lymphocyte ratio (PLR)	> 160	58	3.15	− 8.20, 14.50	0.590
**CPET-derived parameters**					
***i. Continuous***					
• Peak VO_2_ (ml/kg/min)		79	− 0.74	− 1.60, 0.12	0.090
• Peak VO_2_ (ml/min/m^2^) (× 100)		79	− 2.63	− 4.82, − 0.44	0.019
• VO_2_ @ AT (ml/kg/min)		79	− 0.50	− 1.93, 0.93	0.490
• Ve/VCO_2_ @ AT		79	0.63	− 0.09, 1.36	0.088
• P_ET_CO_2_ @ AT (mmHg)		79	− 0.74	− 1.44, − 0.04	0.038
***ii. Dichotomised***	***Risk cut-point***				
• Peak VO_2_ (ml/kg/min)	< 16	79	− 10.20	− 18.90, − 1.53	0.021
• Peak VO_2_ (ml/min/m^2^)	< 710	79	− 11.70	− 20.00, − 3.46	0.005
• VO_2_ @ AT (ml/kg/min)	< 12	79	− 3.74	− 12.60, 5.07	0.410
• Ve/VCO_2_ @ AT	A.1.1.1.1. > 35	79	12.70	0.95, 24.40	0.034
• P_ET_CO_2_ @ AT (mmHg)	< 35	79	− 15.50	− 28.00, − 2.93	0.016
**B. Bi**-**variable analysis**					
• Peak VO_2_ (ml/min/m^2^)	< 710	41	− 15.00	− 24.00, − 5.90	0.001
• Neutrophils (× 10^9^/l)			3.10	0.92, 5.20	0.005
• Neutrophils (× 10^9^/l)	> 7.5	5	22.00	5.20, 39.00	0.010
• Peak VO_2_ (ml/min/m^2^)	< 710	41	− 13.00	− 23.00, − 4.00	0.005
• Neutrophils (× 10^9^/l)	> 7.5	5	24.00	6.60, 42.00	0.007
• Ve/VCO_2_ @ AT	> 35	12	7.30	− 5.80, 21.00	0.280
• Heart rate change: baseline to peak (beats per minute)	< 25	76	− 30.00	− 51.00, − 9.20	0.005
• Peak VO_2_ (ml/kg/min)	< 710	41	− 9.30	− 17.00, − 1.20	0.024

*CPET*, cardiopulmonary exercise testing; *Peak VO*_*2*_, maximum rate of oxygen consumption measured during incremental exercise; *VO*_*2*_ *@ AT*, oxygen uptake at anaerobic threshold; Ve/VCO_2_, *minute ventilation relative to carbon dioxide elimination; P*_*ET*_*CO*_*2*_ *@ AT*, partial pressure of end tidal carbon dioxide at anaerobic threshold

The Charlson Co-morbidity Index was a poor discriminator of postoperative complications. In contrast, low preoperative haemoglobin and albumin (potential modifiable risk factors) were associated with increased risk for postoperative complications. A preoperative pro-inflammatory state, depicted by a high preoperative neutrophil count (absolute or dichotomised at 7.5 × 10^9^ cells/L), had a strong association with increased postoperative complications. This predictive value was significantly (*P* < 0.01) additive when preoperative neutrophil count was combined with albumin (a modification to the Glasgow Prognostic Score) (He et al., 2018) or considered within a bi-variable model with the CPET-derived parameter pVO_2_ (corrected to body surface area).

CPET-derived gas exchange parameters that were most predictive of postoperative complications included pVO_2_ (corrected to body surface area) and the measures of ventilation-perfusion (VQ) matching, namely low partial pressure of end-tidal CO_2_ (P_ET_CO_2_) and high ventilatory inefficiency (Ve/VCO_2_) both measured at anaerobic threshold. Peak VO_2_ (corrected to body surface area) and chronotropic response (heart rate > 25 beats per minute increase during exercise from rest to peak VO_2_) had additive predictive value within a bi-variable model.

### Days at Home within 90 days after surgery

Univariable linear regression assessed the association between Charlson Co-morbidity Index, traditional preoperative laboratory values, CPET-derived variables, and Days at Home within 90 days after surgery (DAH-90; Table 3).

**Table 3:** Days at Home within 90 days after surgery (DAH-90). Association between the DAH-90, a patient centric measure, and continuous or dichotomised preoperative variables, including the Charlson Co-morbidity score, laboratory biomarkers and CPET-derived variables. Analysis was done using linear regression analysis

Preoperative parameters	Dichotomised reference level	***N***	beta	95%CI	***P***-value
**A. Univariable analysis**					
**Charlson Co-morbidity Index**					
***i. Continuous***		80	− 0.86	− 2.59, 0.86	0.330
***ii. Dichotomised***	< 5	80	− 4.47	− 12.10, 3.14	0.250
**Laboratory parameters**					
***i. Continuous***					
• Haemoglobin (g/l)		58	0.24	− 0.01, 0.49	0.060
• White cell count (× 10^9^/l)		58	− 1.94	− 3.67, − 0.21	0.028
• Neutrophils (× 10^9^/l)		58	− 2.59	− 4.63, − 0.55	0.013
• Albumin (g/l)		34	1.37	0.57, 2.17	0.001
• Modified (neutrophil-albumin) Glasgow Prognostic Score		34	− 12.50	− 19.40, − 5.48	< 0.001
***ii. Dichotomised***	***Risk cut-point***				
• Haemoglobin (g/l)	< 110	58	10.6	0.09, 21.00	0.048
• White Cell Count (× 10^9^/l	> 11	58	− 26.50	− 43.40, − 9.55	0.002
• Neutrophils (× 10^9^/l	> 7.5	58	− 26.90	− 41.80, − 11.90	< 0.001
• Platelets (× 10^9^/l	> 350	58	− 6.27	− 19.60, 7.07	0.360
• Albumin (g/l)	< 35	34	10.3	0.02, 20.60	0.050
***iii. Ratios***					
Neutrophil-lymphocyte ratio (NLR)	> 4	58	− 0.32	− 9.96, 9.33	0.950
Platelet-lymphocyte ratio (PLR)	> 160	58	− 2.76	− 12.70, 7.24	0.590
**CPET-derived parameters**					
***i. Continuous***					
• Peak VO_2_ (ml/kg/min)		80	0.62	− 0.15, 1.39	0.110
• Peak VO_2_ (ml/min/m^2^) (× 100)		80	2.07	0.12, 4.01	0.038
• VO_2_ @ AT (ml/kg/min)		80	0.45	− 0.81, 1.71	0.490
• Ve/VCO_2_ @ AT		80	− 0.56	− 1.21, 0.08	0.088
• P_ET_CO_2_ @ AT (mmHg)		80	0.68	0.05, 1.31	0.033
***ii. Dichotomised***	***Risk cut-point***				
• Peak VO_2_ (ml/kg/min)	≤ 16	80	10.60	3.00, 18.20	0.006
• Peak VO_2_ (ml/min/m^2^)	< 710	80	9.46	2.08, 16.90	0.012
• VO_2_ @ AT (ml/kg/min)	< 12	80	1.94	− 5.88, 9.75	0.630
• Ve/VCO_2_ @ AT	> 35	80	− 13.3	− 23.7, − 3.01	0.011
• P_ET_CO_2_ @ AT (mmHg)	< 35	80	11.20	− 0.08, 22.60	0.052
**B. Bi-variable analysis**					
• Peak VO_2_ (ml/kg/min)	≤ 16	51	19.00	11.00, 27.00	< 0.001
• Modified (neutrophils-albumin) Glasgow Prognostic Score			− 10.00	− 16.00, − 4.50	< 0.001

*CPET*, cardiopulmonary exercise testing; *Peak VO*_*2*_, maximum rate of oxygen consumption measured during incremental exercise; *VO*_*2*_ *@ AT*, oxygen uptake at anaerobic threshold; *Ve/VCO*_*2*_, *minute ventilation relative to carbon dioxide elimination; P*_*ET*_*CO*_*2*_ *@ AT*, partial pressure of end tidal carbon dioxide at anaerobic threshold

The Charlson Co-morbidity Index did not discriminate low versus high DAH-90. In contrast, low haemoglobin and albumin were associated with reduced DAH-90. A preoperative pro-inflammatory state, depicted by a high preoperative neutrophil count (absolute or dichotomised), was also strongly associated with reduced DAH-90. This was also evident when preoperative inflammation was considered in conjunction with albumin (modified Glasgow Prognostic Score) (*P* < 0.001).

The CPET-derived gas exchange parameters that were most predictive of reduced DAH-90 included increased high ventilatory inefficiency (Ve/VCO_2_) measured at anaerobic threshold and peak VO_2_ (corrected to body weight and to body surface area). Within a bi-variable model, the modified Glasgow Prognostic Score (using neutrophil count) and the CPET-derived parameter pVO_2_ (corrected to body weight) had significant additive predictive value for reduced DAH-90 after surgery (*P* < 0.001).

### Overall survival

Univariable linear regression assessed the association between Charlson Co-morbidity Index, traditional preoperative laboratory values, CPET-derived variables, and overall survival within two years after surgery (Table 4).

**Table 4:** Survival. Association between overall survival and continuous or dichotomised preoperative variables, including Charlson Co-morbidity score, laboratory biomarkers and CPET-derived variables. Analysis was done using Cox proportional hazards regression analysis

Preoperative parameters	Dichotomised reference level	***N***	Hazard ratio or (1/hazard ratio)^**a**^	95%CI	***P***-value
**A. Univariable Analysis**					
**Charlson Co-morbidity Risk Index**					
***i. Continuous***		82	1.03	0.81, 1.32	0.810
***ii. Dichotomised***	< 5	82	1.62	0.54, 4.84	0.380
**Laboratory parameters**					
***i. Continuous***					
• Haemoglobin (g/l)		58	0.93, ^a^1.08	0.89, 0.98	0.001
• White cell count (× 10^9^/l)		58	1.29	1.08, 1.55	0.012
• Neutrophils (× 10^9^/l)		58	1.36	1.10, 1.67	0.010
• Albumin (g/l)		34	0.89, ^a^1.12	0.80, 1.00	0.050
• Modified (neutrophil-albumin) Glasgow Prognostic Score		34	3.16	1.21, 8.27	0.024
***ii. Dichotomised***	***Risk cut-point***				
• Haemoglobin (g/l)	<110	58	0.232, ^a^ 4.31	0.06, 0.87	0.032
• White Cell Count (× 10^9^/l)	> 11	58	4.40	0.91, 21.30	0.110
• Neutrophils (× 10^9^/l)	> 7.5	58	6.79	1.68, 27.50	0.020
• Platelets (× 10^9^/l)	> 350	58	2.98	0.74, 11.90	0.150
• Albumin (g/l)	< 35	34	0.22, ^a^4.55	0.04, 1.12	0.051
***iii. Ratios***					
Neutrophil-lymphocyte ratio (NLR)	> 4	58	1.57	0.42, 5.84	0.510
Platelet-lymphocyte ratio (PLR)	> 160	58	0.92	0.23, 3.67	0.900
**CPET-derived parameters**					
***i. Continuous***					
• Peak VO_2_ (ml/kg/min)		82	0.91, ^a^1.10	0.80, 1.04	0.150
• Peak VO_2_ (ml/min/m^2^) (× 100)		82	0.65, ^a^1.54	0.45, 0.94	0.012
• VO_2_ @ AT (ml/kg/min)		82	0.89, ^a^1.12	0.72, 1.10	0.250
• Ve/VCO_2_ @ AT		82	1.09	1.02, 1.15	0.014
• P_ET_CO_2_ @ AT (mmHg)		82	0.89, ^a^1.12	0.82, 0.96	0.004
***ii. Dichotomised***	***Risk cut-point***				
• Peak VO_2_ (ml/kg/min)	< 16	82	0.35, ^a^ 2.86	0.12, 1.00	0.049
• Peak VO_2_ (ml/min/m^2^)	< 710	82	0.13, ^a^7.70	0.03, 0.56	0.001
• VO_2_ @ AT (ml/kg/min)	< 12	82	0.69, ^a^1.45	0.23, 2.05	0.490
• Ve/VCO_2_ @ AT	> 35	82	6.12	2.13, 17.50	0.001
• P_ET_CO_2_ @ AT (mmHg)	< 35	82	0.160, ^a^6.25	0.05, 0.47	0.002
**B. Bi-variable analysis**					
• Haemoglobin (g/l)			0.94, ^a^1.06	0.90, 0.99	0.017
• Ve/VCO_2_ @ AT	>35	10	2.60	0.63, 11.00	0.180
• Peak VO_2_ (ml/min/m^2^)	<710	42	0.18	0.04, 0.85	0.013
• Ve/VCO_2_ @ AT	> 35	13	^a^5.56, 3.70	1.30, 11.00	0.020
• Peak VO_2_ (ml/min/m^2^)	> 710	28	0.11, ^a^9.09	0.01, 0.87	0.007
• Neutrophils (× 10^9^/l)			1.40	1.10, 1.70	0.008

*CPET*, cardiopulmonary exercise testing; *Peak VO*_*2*_, maximum rate of oxygen consumption measured during incremental exercise; *VO*_*2*_ *@ AT*, oxygen uptake at anaerobic threshold; *Ve/VCO*_*2*_, *minute ventilation relative to carbon dioxide elimination; P*_*ET*_*CO*_*2*_ *@ AT*, partial pressure of end tidal carbon dioxide at anaerobic threshold

^a^Inverse of hazard ratio to show directional change related to overall survival relative to other variables

The Charlson Co-morbidity Index did not discriminate survivors from non-survivors. In contrast, low haemoglobin was strongly associated with reduced overall survival. Similarly, a preoperative pro-inflammatory state, depicted by a high preoperative neutrophil count (absolute or dichotomised), discriminated overall survival.

**Fig. 1:**
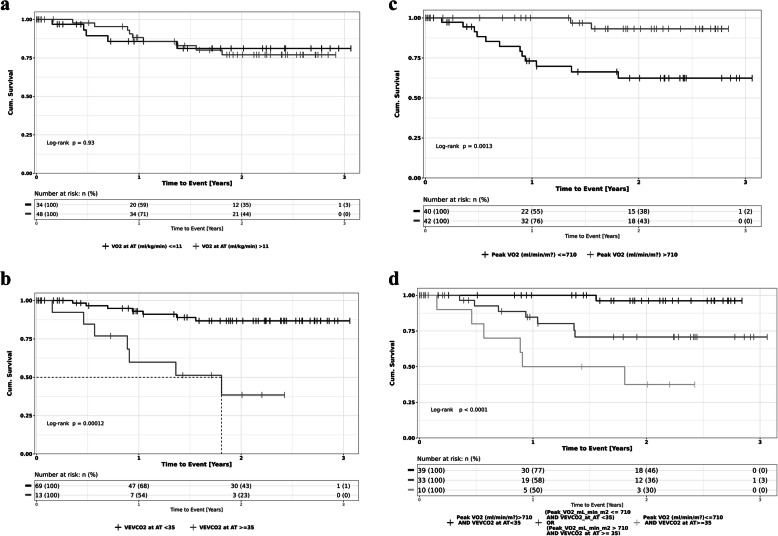
Kaplan-Meier curves showing survival based on CPET-derived gas exchange parameters—VO_2_ at AT (dichotomised at AT > 11 ml/kg/min; Log-Rank Mantel-Cox = not significant), Ve/VCO_2_ at AT (dichotomised at Ve/VCO2 < 35; Log-Rank Mantel-Cox; *P* = 0.001), VO_2_ at peak exercise (dichotomised at pVO2 >710 ml/min/m^2^; Log-Rank Mantel-Cox; *P* < 0.001) and when Ve/VCO_2_ and peak VO_2_ variables are combined. **a** Univariate of VO_2_ (ml/kg/min) at AT. **b** Ve/VCO_2_ at AT. **c** Univariate of peak VO_2_ (ml/min/m^2^) corrected to body surface area. **d** Bivariate of Ve/VCO_2_ and peak VO_2_ corrected to body surface area

CPET-derived gas exchange parameters that were most predictive of reduced overall survival included poor CO_2_ elimination at AT, namely low partial pressure for end-tidal CO_2_ and high ventilatory inefficiency (Ve/VCO_2_) at anaerobic threshold, and pVO_2_ (corrected to body surface area). Comparison of Kaplan-Meier curves (Fig. 1a–d) showed significant difference in survival when P_ET_CO_2_ at AT was dichotomised at 35 mmHg (*P* = 0.001), when Ve**/**VCO_2_ was dichotomised at 35 (*P* = 0.001), when pVO_2_ was dichotomised at 710 ml/min/m^2^ (*P* < 0.01), and when Ve**/**VCO2 and peak VO2 were considered together. Importantly, VO_2_ at AT (dichotomised at 11 ml/kg/min) did not discriminate survivors from non-survivors.

Within bivariate modelling, when inflammation was considered in conjunction with albumin (a modification to the Glasgow Prognostic Score) or neutrophil count considered with peak VO_2_ (corrected to body weight) it added significant ability to discriminate between survivors and non-survivors. Similarly, when ventilatory efficiency (Ve/VCO_2_) was considered within a bi-variable model with the CPET-derived parameter peak VO_2_ (corrected to body weight), it also added significant predictive value for reduced survival after surgery (Fig. 1; *p* < 0.0001).

### Exploratory CPET parameters

Heart rate at peak exercise and the heart rate recovery (HRR) after peak exercise are higher among the survivors than non-survivors. While change in heart rate and change in VO_2_ over the entire testing period were not statistically different between survivors and non-survivors, the recovery phase slopes for HRR and for oxygen consumption (VO_2_R) were significantly different between survivors and non-survivors (Table 5) with modest ability to predict survival (HRR: AUROC 0.74, 95% CI 0.59–0.89; *P* = 0.002 and HRR > 0.87 had 69% sensitivity and 81% specificity; VO_2_R: 0.74, 95% CI 0.61-0.86; *P* = 0.008 and VO_2_R > 0.57 had 85% sensitivity and 66% specificity). The recovery phase slopes for CPET-derived CO_2_ exchange parameters (VCO_2_, P_ET_CO_2_, Ve/VCO_2_) were not significantly different between survivors and non-survivors.

**Table 5:** CPET-derived recovery phase parameters, with difference between survivors and non-survivors after major colorectal cancer surgery at 1-year follow-up

Variables	Survivors	Non-survivors	AUROC	***P***-value
VO_2_: slope (IQR)	0.54 (0.46-0.62)	0.61 (0.57-0.70)	0.74	0.008
Heart rate: slope (IQR)	0.81 (0.74-0.87)	0.89 (0.81-0.95)	0.74	0.006
Ve/VCO_2_: slope (IQR)	28.5 (23.90-32.90)	32.1 (24.20-39.40)	0.59	0.318
P_ET_CO_2_: slope (IQR)	0.95 (0.92-1.00)	0.97 (0.92-1.10)	0.60	0.279

Results are presented as median with IQR

A larger ‘Ve/VCO_2_ slope’ implies a slower recovery from peak performance to baseline

*VO*_*2*_, maximum rate of oxygen consumption measured during incremental exercise; *Ve/VCO*_*2*_, *minute ventilation relationship to carbon dioxide elimination; P*_*ET*_*CO*_*2*_, end tidal partial pressure of carbon dioxide

## Discussion

This study demonstrates that a ‘triple low’ state, characterised by low haemoglobin (anaemia), low albumin, and low functional capacity compounded by a pro-inflammatory state, as assessed by routine inflammatory markers are associated with poorer postoperative outcomes, including medium-term overall survival. These findings add to the emerging body of evidence that dynamic assessment of physiological fitness using CPET can help predict adverse postoperative outcomes (including post-operative complications and mortality) in patients undergoing major colorectal cancer surgery. The Charlson Co-morbidity Index appears unreliable for predicting post-operative complications in this patient population.

### Utilisation of CPET parameters

Previous studies have demonstrated that patients undergoing intra-abdominal surgery with an AT between 10 and 12 ml/kg/min have increased postoperative risk, with an AT of < 10.1 ml/kg/min being a strong predictor of morbidity, and an AT < 10.9 ml/kg/min being a predictor of mortality (Moran et al., 2016). In a recent meta-analysis of objective assessment of physical fitness in patients undergoing colorectal cancer surgery we reported that deconditioned (AT < 11 ml/kg/min) patients had a three- to five-fold higher incidence of postoperative complications than those patients deemed ‘fit’, but we were unable to identify a pooled cut point to predict postoperative mortality (Lee et al., 2018). While pVO_2_ has been demonstrated to be an independent predictor of mortality (Jones et al., 2011), with significant risk of perioperative complications reported with a pVO_2_ of < 15 ml/kg/min (Smith et al., 2009), a recent international prospective cohort study (the METS study) suggested that a low AT or low pVO_2_ did not predict for a composite of postoperative cardiac complications and mortality (Wijeysundera et al., 2018). This same study, however, confirmed the value of peak VO_2_ as a bi-variate metric to be predictive of all-cause non-cardiac complications after surgery in a mixed cohort of surgical patients with relative low preoperative risk (Wijeysundera et al., 2018). In our study cohort of patients having major colorectal cancer surgery, we were unable to demonstrate significant association between VO_2_ kinetics at AT and adverse postoperative outcomes. The strongest association between postoperative mortality in our study was seen with VO_2_ at peak exercise that was adjusted to body surface area (rather than weight) and for CO_2_ kinetics at AT (Ve/VCO_2_ or P_ET_CO_2_ at AT). In this small cohort, one in two patients with preoperative pVO_2_ < 710 ml/min/m^2^ and Ve/VCO_2_ at AT > 35 had died within one year of surgery (Fig. 1d).

Nagamatsu et al. reported a similar association between pVO2 adjusted for body surface area and postoperative complications in patients having an oesophagectomy (Nagamatsu et al., 2001; Nagamatsu et al., 1994). Indexing VO_2_ to body surface area rather than body weight was performed in order to minimise variability due to extremes of body weight. There is emerging recognition that sarcopenic obesity (obesity with depleted muscle mass), caused by cancer, as well as toxicity of chemotherapy, may be predictive of disease-specific morbidity and mortality (Prado et al., 2008). Our patient cohort included patients with recurrent, or advanced gastrointestinal cancer, commonly having multiple courses of preoperative chemotherapy and potential sarcopenia, making it necessary to index CPET derived variables to body surface area rather than absolute body weight.

### Utilisation of preoperative biomarkers

Statistical association of low haemoglobin and low albumin with postoperative complications and overall survival allude to additional modifiable risk factors that may not only add value to CPET-derived risk prediction but also underpin the need for haematinic and nutritional optimisation within the setting of prehabilitation. The concept of using perioperative biomarkers to stratify surgical risk has its foundations in gauging the risk of postoperative cardiac complications (Edwards et al., 2011). Optimisation of the oxygen-carrying capacity of the surgical patient by addressing anaemia has been shown to increase AT and pVO_2 (__Agostoni et al.,_ 2010_)_. Both cancer biology and anticancer therapy are associated with a pro-inflammatory state (de Visser et al., 2006) and relationships between different haematological markers have been previously used to prognosticate cancer risk. Specifically, platelet/lymphocyte (PLR), lymphocyte/monocyte (LMR) and neutrophil/lymphocyte (NLR) ratios have been investigated as preoperative risk indicators (Guo et al., 2017; Templeton et al., 2014; Chan et al., 2017). It has been demonstrated that in patients with operable colorectal cancer, PLR was associated with poor prognosis, whereas LMR was associated with increased overall survival for patients undergoing curative colorectal resections (Guo et al., 2017; Chan et al., 2017). While the ratios of these haematological markers did not demonstrate an association with the postoperative outcomes of interest in our patient population, there was statistically significant association with preoperative neutrophil count, signalling the association between preoperative inflammation and post-operative outcomes. Though our sample sizes were limited for biomarker analysis (haemoglobin/neutrophil count: *n* = 58; albumin count: *n* = 34), larger prospective studies assessing multiple variables alongside CPET are needed to delineate modifiable risk factors that may benefit from intervention preoperatively, within the setting of a multimodal prehabilitation program.

### Exploratory CPET recovery parameters

Ackland et al. have demonstrated that heart rate recovery (HRR) has good predictive capability for morbidity within five days of surgery (Ackland et al., 2019). We similarly demonstrate that heart rate kinetics during preoperative CPET testing has a modest ability to predict intermediate-term mortality following major intra-abdominal cancer surgery. HRR is an independent predictor for 6-year all-cause mortality in the epidemiological (non-surgical) population (Cole et al., 1999). Patients with prolonged HRR were more likely to be elderly and also possess cardiac risk factors (Simões et al., n.d.). Furthermore, large studies examining healthy patients without cardiovascular disease have also reported that HRR is directly linked to mortality (Cole et al., 2000; Nishime et al., 2000; Shetler et al., 2001).

Our findings that HRR is a prognostic factor, as a reflection of cardiovascular reserve, or a predictor of autonomic dysfunction (Imai et al., 1994; Perini et al., 1989), is supported by studies showing HRR is associated perioperative morbidity (Ackland et al., 2015; Ackland et al., 2018), improved survival in conditions such as bacterial peritonitis, hypovolemic shock, and myocardial ischaemia (Guarini et al., 2003; Boland et al., 2011; Mioni et al., 2005). Taking all the existing evidence together, HRR measured during the recovery phase of CPET has the potential to be an important marker of parasympathetic activity and a risk predictor of perioperative morbidity and mortality risk and should be further investigated.

### Limitations of this study

This cohort reflects a gastrointestinal surgical population in a quaternary institution with multiple variables that may affect outcomes and these results may have limited applicability to other major oncological subspecialty procedures. The size of this cohort was limited and therefore some of our negative findings may reflect the power of the study. Given the exploratory nature of this retrospective study, we have restricted our focus to comorbid disease and have a limited description of intraoperative variables. The statistical power of the results may be reduced due to the number of analysed variables. Furthermore, while referral to CPET in the study centre is based on hospital guidelines, the pattern of referral could have introduced an element of selection bias.

## Conclusion

Patients presenting with a ‘triple low’ preoperative state (anaemia {low haemoglobin}, malnutrition {low albumin}, and deconditioned {low functional capacity, e.g. low peak VO2}) are at greatest risk of postoperative complications and death. This is further compounded by a pro-inflammatory state. This study demonstrates that in complex colorectal cancer patients undergoing major cancer surgery, one in two patients with a preoperative pVO_2_ < 710 ml/kg/m^2^ and Ve/VCO_2_ at AT > 35 or P_ET_CO_2_ at AT < 35 mmHg will die within 1 year of surgery. In light of this, the current focus on conventional CPET parameters such as VO_2_ at AT and peak VO_2_ should be superseded by a holistic approach that analyses multiple physiological and biochemical parameters. This will not only improve risk prediction but to identify opportunity to optimise reversible patient factors within the prehabilitation window. Large, prospective multivariate trials are required to expand our understanding of modifiable risk factors and guide preoperative optimisation prior to major cancer surgery.

## Data Availability

Please contact author for data request.

## Abbreviations

AT: Anaerobic threshold
CPET: Cardiopulmonary exercise testing
CCI: Comprehensive Complication Index
DAH-90: Days at Home within 90 days
DASI: Duke Activity Status Index
HRR: Heart rate recovery
NLR: Neutrophil-lymphocyte ratio
PLR: Platelet-lymphocyte ratio

## References

[CR1] Ackland GL, Abbott TEF, Minto G (2019). Heart rate recovery and morbidity after noncardiac surgery: Planned secondary analysis of two prospective, multi-centre, blinded observational studies. PLoS One.

[CR2] Ackland GL, Iqbal S, Paredes LG (2015). Individualised oxygen delivery targeted haemodynamic therapy in high-risk surgical patients: a multicentre, randomised, double-blind, controlled, mechanistic trial. Lancet Respir Med.

[CR3] Ackland GL, Minto G, Clark M (2018). Autonomic regulation of systemic inflammation in humans: a multi-center, blinded observational cohort study. Brain Behav Immun.

[CR4] Agostoni P, Salvioni E, Debenedetti C (2010). Relationship of resting hemoglobin concentration to peak oxygen uptake in heart failure patients. Am J Hematol.

[CR5] Bainbridge D, Martin J, Arango M, Cheng D (2012). Perioperative and anaesthetic-related mortality in developed and developing countries: a systematic review and meta-analysis. Lancet.

[CR6] Barberan-Garcia A, Ubré M, Roca J (2018). Personalised prehabilitation in high-risk patients undergoing elective major abdominal surgery : a randomized blinded controlled trial. Ann Surg.

[CR7] Bilimoria KY, Liu Y, Paruch JL (2013). Development and evaluation of the universal ACS NSQIP surgical risk calculator: a decision aide and informed consent tool for patients and surgeons. J Am Coll Surg.

[CR8] Boland C, Collet V, Laterre E, Lecuivre C, Wittebole X, Laterre P-F (2011). Electrical vagus nerve stimulation and nicotine effects in peritonitis-induced acute lung injury in rats. Inflammation.

[CR9] Bolshinsky V, Li MH, Ismail H, Burbury K, Riedel B, Heriot A (2018). Multimodal prehabilitation programs as a bundle of care in gastrointestinal cancer surgery: a systematic review. Dis Colon Rectum.

[CR10] Boney O, Bell M, Bell N (2015). Identifying research priorities in anaesthesia and perioperative care: final report of the joint National Institute of Academic Anaesthesia/James Lind Alliance Research Priority Setting Partnership. BMJ Open.

[CR11] Chan JCY, Chan DL, Diakos CI (2017). The lymphocyte-to-monocyte ratio is a superior predictor of overall survival in comparison to established biomarkers of resectable colorectal cancer. Ann Surg.

[CR12] Chang C-M, Yin W-Y, Wei C-K (2016). Adjusted age-adjusted Charlson Comorbidity Index Score as a risk measure of perioperative mortality before cancer surgery. PLoS One.

[CR13] Chen N, Li W, Huang K (2017). Increased platelet-lymphocyte ratio closely relates to inferior clinical features and worse long-term survival in both resected and metastatic colorectal cancer: an updated systematic review and meta-analysis of 24 studies. Oncotarget.

[CR14] Cole CR, Blackstone EH, Pashkow FJ, Snader CE, Lauer MS (1999). Heart-rate recovery immediately after exercise as a predictor of mortality. N Engl J Med.

[CR15] Cole CR, Foody JM, Blackstone EH, Lauer MS (2000). Heart rate recovery after submaximal exercise testing as a predictor of mortality in a cardiovascularly healthy cohort. Ann Intern Med.

[CR16] de Visser KE, Eichten A, Coussens LM (2006). Paradoxical roles of the immune system during cancer development. Nat Rev Cancer.

[CR17] Dindo D, Demartines N, Clavien PA. Classification of surgical complications: a new proposal with evaluation in a cohort of 6336 patients and results of a survey. Ann Surg. 2004;240. 10.1097/01.sla.0000133083.54934.ae. 10.1097/01.sla.0000133083.54934.ae PMC1360123 15273542

[CR18] Edwards M, Whittle J, Ackland GL (2011). Biomarkers to guide perioperative management. Postgrad Med J.

[CR19] Findlay GP, Protopapa KL, Smith NCE, Mason M, GAPL (2011). Knowing the Risk: a review of the peri-operative care of surgical patients. National Confidential Enquiry into Patient Outcome and Death.

[CR20] Guarini S, Altavilla D, Cainazzo M-M (2003). Efferent vagal fibre stimulation blunts nuclear factor-kappaB activation and protects against hypovolemic hemorrhagic shock. Circulation..

[CR21] Guo Y-H, Sun H-F, Zhang Y-B (2017). The clinical use of the platelet/lymphocyte ratio and lymphocyte/monocyte ratio as prognostic predictors in colorectal cancer: a meta-analysis. Oncotarget.

[CR22] He L, Li H, Cai J (2018). Prognostic value of the Glasgow prognostic score or modified Glasgow prognostic score for patients with colorectal cancer receiving various treatments: a systematic review and meta-analysis. Cell Physiol Biochem.

[CR23] Hightower CE, Riedel BJ, Feig BW (2010). A pilot study evaluating predictors of postoperative outcomes after major abdominal surgery: physiological capacity compared with the ASA physical status classification system. Br J Anaesth.

[CR24] Howard R, Yin YS, McCandless L, Wang S, Englesbe M, Machado-Aranda D (2019). Taking control of your surgery: impact of a prehabilitation program on major abdominal surgery. J Am Coll Surg.

[CR25] Imai K, Sato H, Hori M (1994). Vagally mediated heart rate recovery after exercise is accelerated in athletes but blunted in patients with chronic heart failure. J Am Coll Cardiol.

[CR26] Jack S, West M, Grocott MP (2011). Perioperative exercise training in elderly subjects. Best Pr Res Clin Anaesthesiol.

[CR27] Jones LW, Liang Y, Pituskin EN (2011). Effect of exercise training on peak oxygen consumption in patients with cancer: a meta-analysis. Oncologist.

[CR28] Lee CHA, Kong JC, Ismail H, Riedel B, Heriot A (2018). Systematic review and meta-analysis of objective assessment of physical fitness in patients undergoing colorectal cancer surgery. Dis Colon Rectum.

[CR29] Levett DZ, Grocott M, Miller TE, Scott MJ (2015). Cardiopulmonary exercise testing for risk prediction in major abdominal surgery.

[CR30] Levett DZH, Jack S, Swart M (2018). Perioperative cardiopulmonary exercise testing (CPET): consensus clinical guidelines on indications, organization, conduct, and physiological interpretation. Br J Anaesth.

[CR31] Mioni C, Bazzani C, Giuliani D (2005). Activation of an efferent cholinergic pathway produces strong protection against myocardial ischemia/reperfusion injury in rats. Crit Care Med.

[CR32] Moonesinghe SR, Mythen MG, Das P, Rowan KM, Grocott MP (2013). Risk stratification tools for predicting morbidity and mortality in adult patients undergoing major surgery: qualitative systematic review. Anesthesiology.

[CR33] Moran J, Wilson F, Guinan E, McCormick P, Hussey J, Moriarty J (2016). Role of cardiopulmonary exercise testing as a risk-assessment method in patients undergoing intra-abdominal surgery: a systematic review. Br J Anaesth.

[CR34] Myles PS, Grocott MPW, Boney O, Moonesinghe SR (2016). Standardizing end points in perioperative trials: towards a core and extended outcome set. Br J Anaesth.

[CR35] Nagamatsu Y, Shima I, Yamana H, Fujita H, Shirouzu K, Ishitake T (2001). Preoperative evaluation of cardiopulmonary reserve with the use of expired gas analysis during exercise testing in patients with squamous cell carcinoma of the thoracic esophagus. J Thorac Cardiovasc Surg.

[CR36] Nagamatsu Y, Yamana H, Fujita H (1994). The simultaneous evaluation of preoperative cardiopulmonary functions of esophageal cancer patients in the analysis of expired gas with exercise testing. Nihon Kyobu Geka Gakkai Zasshi.

[CR37] Nishime EO, Cole CR, Blackstone EH, Pashkow FJ, Lauer MS (2000). Heart rate recovery and treadmill exercise score as predictors of mortality in patients referred for exercise ECG. JAMA.

[CR38] Perini R, Orizio C, Comandè A, Castellano M, Beschi M, Veicsteinas A (1989). Plasma norepinephrine and heart rate dynamics during recovery from submaximal exercise in man. Eur J Appl Physiol Occup Physiol.

[CR39] Prado CM, Lieffers JR, McCargar LJ (2008). Prevalence and clinical implications of sarcopenic obesity in patients with solid tumours of the respiratory and gastrointestinal tracts: a population-based study. Lancet Oncol.

[CR40] R Z (2021). Neutrophil-to-lymphocyte ratio, past, present and future perspectives. Bratisl Lek Listy.

[CR41] Shetler K, Marcus R, Froelicher VF (2001). Heart rate recovery: validation and methodologic issues. J Am Coll Cardiol.

[CR42] Silver JK, Baima J (2013). Cancer prehabilitation: an opportunity to decrease treatment-related morbidity, increase cancer treatment options, and improve physical and psychological health outcomes. Am J Phys Med Rehabil.

[CR43] Simões RP, Bonjorno JC, Beltrame T, Catai AM, Arena R, Borghi-Silva A. Slower heart rate and oxygen consumption kinetic responses in the on- and off-transient during a discontinuous incremental exercise: effects of aging. Brazilian J Phys Ther. 17(1):69–76 http://www.ncbi.nlm.nih.gov/pubmed/23117650. Accessed 16 July 2018. 10.1590/s1413-35552012005000056 23117650

[CR44] Slankamenac K, Graf R, Barkun J, Puhan MA, Clavien PA (2013). The comprehensive complication index: a novel continuous scale to measure surgical morbidity. Ann Surg.

[CR45] Smith TB, Stonell C, Purkayastha S, Paraskevas P (2009). Cardiopulmonary exercise testing as a risk assessment method in non cardio-pulmonary surgery: a systematic review. Anaesthesia.

[CR46] Templeton AJ, MG MN, Šeruga B, et al. Prognostic role of neutrophil-to-lymphocyte ratio in solid tumors: a systematic review and meta-analysis. JNCI J Natl Cancer Inst. 2014;106(6). 10.1093/jnci/dju124. 10.1093/jnci/dju124 24875653

[CR47] Tsimopoulou I, Pasquali S, Howard R (2015). Psychological prehabilitation before cancer surgery: a systematic review. Ann Surg Oncol.

[CR48] Vardy JL, Dhillon HM, Pond GR, Renton C, Clarke SJ, Tannock IF (2018). Prognostic indices of inflammatory markers, cognitive function and fatigue for survival in patients with localised colorectal cancer. ESMO open.

[CR49] West MA, Loughney L, Lythgoe D (2014). The effect of neoadjuvant chemoradiotherapy on whole-body physical fitness and skeletal muscle mitochondrial oxidative phosphorylation in vivo in locally advanced rectal cancer patients--an observational pilot study. PLoS One.

[CR50] Wijeysundera DN, Pearse RM, Shulman MA, et al. Assessment of functional capacity before major non-cardiac surgery: an international, prospective cohort study. www.thelancet.com. Published online 2018. 10.1016/S0140-6736(18)31131-0 10.1016/S0140-6736(18)31131-0 30070222

